# Screening for Distress in Oncological Patients: The Revised Version of the Psychological Distress Inventory (PDI-R)

**DOI:** 10.3389/fpsyg.2022.859478

**Published:** 2022-05-06

**Authors:** Alessandro Alberto Rossi, Maria Marconi, Federica Taccini, Claudio Verusio, Stefania Mannarini

**Affiliations:** ^1^Section of Applied Psychology, Department of Philosophy, Sociology, Education, and Applied Psychology, University of Padova, Padua, Italy; ^2^Interdepartmental Center for Family Research, University of Padova, Padua, Italy; ^3^Department of Medical Oncology, Presidio Ospedaliero di Saronno, ASST Valle Olona, Saronno, Italy

**Keywords:** distress, palliative care, distress thermometer, psychological distress, oncology, psycho-onchology, psycho-oncological distress, psycho-oncological care

## Abstract

**Background:**

Psychological research in oncological settings is steadily increasing and the construct of psychological distress has rapidly gained popularity—leading to the development of questionnaires aimed at its measurement. The Psychological Distress Inventory (PDI) is one of the most used instruments, but its psychometric properties were not yet deeply evaluated. The present studies aimed at investigating the psychometric properties of the PDI (Study 1) and providing a revised version of the tool (Study 2).

**Methods:**

Oncological outpatients were enrolled at the Department of Medical Oncology of the Presidio Ospedaliero of Saronno, ASST Valle Olona, Italy. For the first study (*N* = 251), an Exploratory Graph Analysis was used to explore the item structure of the PDI. In the second study (*N* = 902), the psychometric properties of the revised PDI (PDI-R) were deeply assessed.

**Results:**

Study 1 showed that the PDI has a not clear structure and it should be reconsidered. On the opposite, Study 2 showed that the revised version (PDI-R) has a solid factorial structure, it is invariant across gender and age, and it has good psychometric properties.

**Conclusion:**

Results suggest that the PDI-R is a reliable measure of psychological distress in different samples of oncological patients, with stronger psychometric properties than the original version. Its use in the clinical and research field is therefore recommended to improve the quality of both assessment and treatment of psychological distress in patients with oncological problems.

## Introduction

Cancer disease is one of the most common chronic diseases (Crocetti et al., 2006; Jemal et al., 2006) and, with about 9.6 million deaths per year (Ferlay et al., 2019; Teo et al., 2019), represents one of the major causes of death worldwide (WHO, 2014). In Italy, in 2019 cancers alone accounted for 29% of deaths, the most frequent types being breast (53,500), colorectal (49,000), lung (42,500), prostate (37,000), and bladder (29,700) (AIRTUM Working Group, 2021).

Despite the advances in the medical field, the diagnosis of cancer brings with it physical changes associated with the disease itself or to treatment side effects and certainly represents a stressful life event that may induce psychological distress in both patients and their loved ones (Norton et al., 2004; Sellick and Edwardson, 2007; Rossi et al., 2021)—also significantly altering the quality of their relationship (Mannarini and Boffo, 2014; Mannarini et al., 2017; Saritas et al., 2017; Catania et al., 2019; Granek et al., 2019; Rakici and Karaman, 2019).

Negative emotions might relate to the threat to life, and the uncertainty for both the treatment outcomes and the entailed suffering (Peck, 1972; Maguire, 1985; Holland, 1992; Amadori et al., 2002; Grassi and Riba, 2012; Wise et al., 2013; Clerici and Veneroni, 2014)—and studies reveal that at least one-third of the individuals diagnosed with cancer report high levels of psychological distress; which increase as the disease worsens (Carlson and Bultz, 2003; Sellick and Edwardson, 2007).

Psychological distress in the oncological setting corresponds to the cancer-related totality of emotions and feelings experienced by patients that may affect their ability to cope with cancer itself (Holland and Bultz, 2007; Compen et al., 2018). Indeed, evidence exists for the association between high levels of distress and decreased quality of life (Derogatis et al., 1983; Maguire, 1985; Fallowfield, 1988; Fallowfield et al., 2001; Riba et al., 2019), adherence to treatment, self-management (Newell et al., 1998; Partridge et al., 2002; Spiegel and Giese-Davis, 2003; Moore, 2010; Granek et al., 2019; Oliveri et al., 2019; Panzeri et al., 2021a), and survival in patients with oncological problems (Trask et al., 2002; Kwak et al., 2013; Ng et al., 2017). Further, Bultz and Carlson (2006) considered psychological distress a fundamental marker of wellbeing and—due to its reciprocal influence with the physical status of the sufferers (Zabora et al., 1997, 2001; Carlson et al., 2004; Louison et al., 2019; Panzeri et al., 2021b; Rossi Ferrario et al., 2021)—a key indicator of the patient’s global health (Bultz and Carlson, 2006) to the point of being listed as the *sixth vital sign* to be investigated in medicine (Grassi and Riba, 2012; Wise et al., 2013; Riba et al., 2019) along with blood pressure, temperature, heart rate, respiratory rate, and pain (Rose and Clarke, 2010; Cutillo et al., 2017).

However, this construct is still is often misinterpreted in literature and confused with other terms, such as «*symptom distress*» (Ridner, 2004)—which corresponds to patients’ discomfort related to the perceptions of their symptoms (McCorkle and Young, 1978). Moreover, it is also seriously underreported in the oncological field (Brain et al., 2006).

Physicians, oncologists, and psycho-oncologists should, therefore, properly screen psychological distress levels in patients with cancer at various (treatment) stages (Carlson and Bultz, 2003; Jacobsen, 2007). To facilitate its measurement, the National Comprehensive Cancer Network^®^ (NCCN^®^) guidelines have indicated that psychological distress may be thought of in terms of (a conjunction of) anxiety and depression (Trask et al., 2002). Massé (2000) has attempted to identify the components of psychological distress and his results show that it consists of a change from a baseline emotional state to the experience of anxiety, depression, aggressiveness, self-depreciation, and demotivation (Massé, 2000). Indeed, when a clinician diagnoses cancer it is generally not possible to make an accurate prognosis; the patient has no guarantee that treatment will restore health. This situation increases the fear of worsening and death. The patient feels hopeless and unable to adopt behaviors useful for the improvement of health. These feelings of total helplessness contribute to the development of depressive symptoms (Rosselli et al., 2015). Furthermore, Pandey et al. (2007) pointed out that anxiety and depression are highly associated with distress in cancer patients to the point of supporting the idea of an overlap between these constructs (Pandey et al., 2007).

Accordingly, studies show that 23.4% of cancer patients report anxiety symptoms which worsen when the cancer symptoms appear, during examinations, and at diagnosis (Stark and House, 2000; Ng et al., 2017; Naser et al., 2021). Moreover, the presence in oncological patients of depressive symptoms ranges from 8 to 24% in non-palliative-care treatment during or after intervention (Krebber et al., 2014). Still, the presence of depressive symptoms might differ according to the cancer type, how these symptoms are evaluated, and the intervention phase—with a higher prevalence in more severe patients (Pirl, 2004; Krebber et al., 2014; Naser et al., 2021). Starting from this background, numerous researches have aimed to create and validate psychological tools for the assessment and measurement of distress in patients with organic pathologies (Carlson and Bultz, 2003; Herschbach et al., 2004; Mitchell, 2010).

However, the Psychological Distress Inventory (PDI, originally developed and validated in Italian context) (Morasso et al., 1996), one of the most worldwide used instruments for the screening and assessment of psychological distress in oncology (Vodermaier et al., 2009; Muzzatti and Annunziata, 2012), was validated without an in-depth evaluation of its psychometric properties, (e.g., factorial structure, measurement invariance, etc.). Indeed, it provides encouraging, albeit incomplete, information on reliability and criterion, concurrent, and discriminant validity but no data on construct validity (Muzzatti and Annunziata, 2012).

Notably, a screening tool that does not have strong and established psychometric properties may lead to misleading results. However, an even more serious problem might be represented by the measurement biases, which may, in turn, lead to underestimation of the measured problem or misdiagnosis—and therefore increase the patient’s suffering (Chad-Friedman et al., 2017). Given the strong impact of distress on health as well as on (also) medical care of cancer patients (DiMatteo et al., 2000), it is therefore essential to have well-established, well-structured, reliable, and that can be used in samples with different characteristics.

Consequently, the first goal (Study 1) of this study is to explore the psychometric properties (i.e., factor dimensionality) of the Psychological Distress Inventory (PDI) (Morasso et al., 1996). On the basis of results of the Study 1, the second aim (Study 2) is to develop and extensively assess the psychometric properties (i.e., structural validity, measurement invariance, screening ability, etc.) of a shortened version of the PDI: the Psychological Distress Inventory—Revised (PDI-R).

## Study 1. Exploring the Dimensional Structure of the Psychological Distress Inventory

### Materials and Methods

#### Procedure

An observational research design was used to investigate the psychological distress experienced by oncological outpatients during the first few weeks between the diagnosis of cancer and the first psychological clinical session—in line with the procedure provided by the HuCARE study protocol (Passalacqua et al., 2016; Marconi et al., 2020; Caminiti et al., 2021; Rossi et al., 2021).

This study was approved by the Ethics Committee of the Ospedale di Saronno (protocol n° 23247). All procedures were in accordance with the ethical standards of the institutional and/or national research committee and with the 1964 Helsinki Declaration and its later amendments or comparable ethical standards.

#### Participants

Oncological outpatients were consecutively recruited from the Department of Medical Oncology, Presidio Ospedaliero di Saronno, ASST Valle Olona, in Saronno (Italy).

Inclusion criteria for participating in the study were: (A) having received a diagnosis of cancer within the last 6 months; (B) being an oncological outpatient (C) not being hospitalized for cancer-related problems within the last year, (D) following intravenous therapy for cancer; (E) being 18 years or older; (F) providing informed consent to participate in the study; and (G) being a native Italian speaker. Exclusion criteria were: (A) inability to understand the items of the questionnaire; (B) impossibility to be assessed due to speaking impairments and/or upcoming medical commitments.

A sample of 270 participants was initially assessed. However, 19 subjects were excluded due to missing data/answers. The final sample comprised, therefore, 251 participants: 120 males (47.80%) and 131 females (52.20%), aged from 20 to 86 years (mean = 63.24, *SD* = 12.56). Considering the type of cancer, 30.7% patients had breast cancer, 27.1% patients had lung cancer, 19.9% patients had gastrointestinal cancer, 10.6% patients had urogenital cancer, 4.2% patients had oncohematological cancer, and 7.5% patients had other type of cancer (e.g., head-and-neck cancer or skin cancer). Considering education level, 22.9% patients had an elementary school diploma, 35.4% patients had a middle school diploma, 34.3% patients had a high school diploma, and 7.4% patients had a bachelor/master’s degree. Considering civil status, 77.2% patients were either in a relationship or married, 11.4% patients were either separated or divorced, 8.3% patients were widowed, and 3.1% patients were single. Considering working status, 50.1% patients were retired, 27.6% patients were dependent workers, 12.4 patients were entrepreneurs, 3.7% patients were housewifes, 4.2 patients were unemployed, and 2% patients declared “other.”

#### Sample Size Determination

The sample size was calculated *a priori* considering the statistical analysis of this study. However, to date, within the framework of Exploratory Graph Analysis (EGA), no “gold standard” rule for determining the minimum sample size required to correctly estimate model parameters seems to exist (Golino and Epskamp, 2017). Therefore, a minimum sample size of 200 individuals was considered adequate.

#### Measures

##### Psychological Distress Inventory

The Psychological Distress Inventory (PDI) was used to measure the degree of psychophysical distress experienced by the sample (Morasso et al., 1996). It represents a self-report questionnaire aimed at detecting distress in individuals suffering from oncological problems by investigating the experience of discomfort related to both emotional and physical domains. The PDI comprises a total of 13 items—with good reliability properties (Morasso et al., 1996)—on a 5-point Likert-type response scale ranging from 1 (*not at all*) to 5 (*very much*). High scores correspond to a high degree of distress perceived by the subject. In this study, Cronbach’s alpha was equal to 0.804 and McDonald’s omega was equal to 0.846.

#### Statistical Analyses

The R software (R Core Team, 2017) was used with the following packages: bootnet (Epskamp et al., 2018); corrplot (Wei and Simko, 2017); EGAnet (Golino and Christensen, 2020), igraph (Csardi and Nepusz, 2006), networkTools (Jones, 2020), qgraph (Epskamp et al., 2012) psych (Revelle, 2018), and psychTools (Revelle, 2020).

Preliminary analyses were performed before carrying out the EGA (Christensen et al., 2020b). First, the normality of items, and the presence of excessive correlations (*r* > 0.70) between items, were inspected (Howell, 2013; Tabachnick and Fidell, 2014). Second, for each item, the level of informativeness was evaluated (Mullarkey et al., 2019; Bottesi et al., 2020). An item should be considered as badly informative as its SD is 2.5 SDs below the average of all the items (Mullarkey et al., 2018, 2019; Marchetti, 2019). Third, item redundancy was checked (Christensen et al., 2020a) by using a Unique Variable Analysis (UVA) approach with weighted topological overlap (wTO) method and adaptive alpha. However, as suggested by existing guidelines, possible item redundancies should be carefully evaluated—for example, considering the aims of the study and/or by inspecting the semantic content of the items (Christensen et al., 2020b).

Consequently, an EGA (Golino and Epskamp, 2017; Christensen et al., 2020b) was performed to assess the item clustering (i.e., dimensionality) of the PDI—given its several advantages over traditional exploratory factor-analytic techniques; it provides greater accuracy in identifying the correct number of factors/dimensions (Golino and Demetriou, 2017; Golino and Epskamp, 2017; Christensen and Golino, 2020; Golino et al., 2020a,b). The EGA produces a plot that might be considered as a “visual guide” (Golino and Epskamp, 2017; Golino et al., 2020a; Panzeri et al., 2021c). It displays the correct number of dimensions—by highlighting which items cluster together and their level of association: the thicker is an edge, the strongest is the relationship between item of a specific cluster (dimension/factor) (Mair, 2018; Christensen and Golino, 2020). The Exploratory Graph Analysis (EGA) was carried out by using a 5,000 parametric bootstrap procedure. Moreover, the GLASSO method with polychoric correlations was used to estimate model parameters (Costantini et al., 2015; Epskamp, 2017; Golino and Epskamp, 2017). In addition, the correct number of dimensions were detected by using the *“Louvain community detection algorithm”* (Blondel et al., 2008). It has demonstrated better performances than the Walktrap algorithm (Pons and Latapy, 2006) in recognizing clusters of items/dimensions (Christensen et al., 2020b).

Once the EGA revealed the number of dimensions composing the PDI, the questionnaire and item statistics were explored. First, item stability (IS) was computed to evaluate the proportion of times the original dimension is exactly replicated across bootstrap resamples—thus, assessesing the occurrence of each item within a certain specific dimension (Christensen et al., 2020b). IS ranging from 0 (“= *perfect instability”*) to 1 (“= *perfect stability”*) and values higher of 0.80 (IS ≥ 0.80) suggest that the item can be considered “stable” and consistently identified in the dimension (Christensen and Golino, 2019). The contribution of each node to the coherence of the dimensions was then assessed using the standardized node strength—namely, network loadings. It is important to note that they represent partial correlation loadings, and the magnitude of these loadings should therefore be interpreted according to the following benchmarks (Christensen and Golino, 2020; Golino et al., 2020c): small: λ_EGA_ ≥ 0.15; moderate: λ_EGA_ ≥ 0.25; large: λ_EGA_ ≥ 0.35.

The internal consistency of each factor was evaluated with Cronbach’s alpha and McDonald’s omega (McDonald, 1999; Howell, 2013).

Lastly, correlations between items were assessed using Pearson’s correlation coefficient and interpreted using Cohen’s (Cohen, 1988) classical benchmarks: *r* < 0.10, trivial; *r* from 0.10 to 0.30, small; *r* from 0.30 to 0.50, moderate; *r* > 0.50, large.

### Results

#### Preliminary Analyses

First, as reported in Table 1, univariate normality was observed for the large majority of the PDI items. Considering non-normal distributed items, only three of them showed small-to-moderate deviations from normality (item#5, item#9, and item#13). Moreover, none of the bivariate correlations exceeded a critical level (*r* ≥ 0.70).

**TABLE 1:** Study 1. Descriptive statistics of items and Exploratory Graph Analysis (EGA) results.

	Descriptive statistics				Item stability (IS)			EGA loadings		
	Mean	*SD*	Skwn.	K	Stab#1	Stab#2	Stab#3	Dim#1 |λ|_EGA_	Dim#2 |λ|_EGA_	Dim#3 |λ|_EGA_
Item#1	1.47	0.776	1.654	2.318	0.001	0.995	0.000	0.082	0.359	0.087
Item#2*	2.97	0.802	–0.137	0.670	0.002	0.000	0.998	–0.049	0.000	0.334
Item#3	2.22	0.976	0.622	–0.010	0.913	0.006	0.001	0.257	0.014	0.000
Item#4	2.49	1.129	0.455	–0.514	0.784	0.063	0.035	0.139	0.053	0.062
Item#5	1.36	0.774	2.413	5.468	0.079	0.888	0.001	0.163	0.231	0.000
Item#6*	2.73	1.030	0.045	–0.146	0.002	0.000	0.998	0.000	0.016	0.302
Item#7	1.86	0.917	0.971	0.617	0.884	0.021	0.001	0.295	0.203	–0.072
Item#8	1.80	1.061	1.349	1.226	0.281	0.140	0.315	0.100	0.019	–0.181
Item#9	1.33	0.667	2.368	6.244	0.954	0.003	0.002	0.273	0.187	–0.069
Item#10	1.53	0.826	1.579	1.987	0.957	0.001	0.001	0.426	0.032	0.096
Item#11	1.46	0.786	1.686	2.018	0.001	0.996	0.000	0.063	0.380	0.089
Item#12	1.97	1.261	1.001	–0.273	0.062	0.635	0.092	0.064	0.167	0.097
Item#13	1.37	0.760	2.344	5.647	0.005	0.986	0.000	0.151	0.272	0.089

*Reverse score item (not reversed); Skwn, Skewness; K, kurtosis; Stability#(…), stability of the item (5,000 replication) on the EGA-based dimension; Dim#(…), EGA-based dimension;| λ| _EGA_, absolute value of the network loading.

Second, the level of informativeness of each item was tested. Results showed that none of the 13 items of the PDI was badly informative (i.e., SD_item_ < 2.5 SD below the mean level of informativeness, M*_SD_* = 1.16 ± 0.18)—suggesting that each item of the PDI provides adequate variability across the sample as well as a good level of informativeness.

Third, item redundancy was inspected. The UVA showed possible redundancy between some items of the PDI. Considering the aim of the study—exploring the structure of the PDI and redefining its psychometric properties—no item was removed. However, item redundancies were carefully considered and deeply studied before setting up Study 2.

#### Exploratory Graph Analysis

**FIGURE 1:**
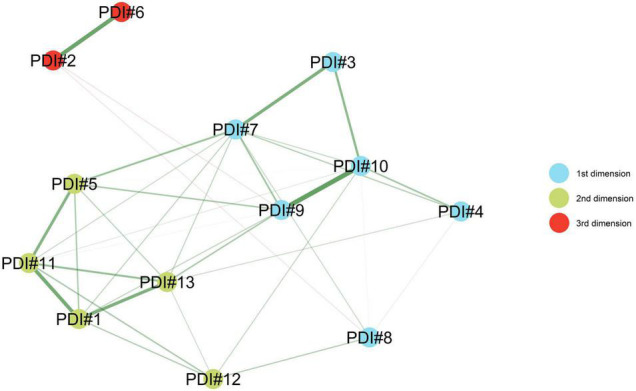
Study 1. Exploratory graph analysis (EGA) of the Psychological Distress Inventory.

As reported in Figure 1 and Table 1, the EGA (5000 bootstrap) clearly identified three dimension/factor solution: median = 3; SE = 0.489; 95%CI [2.040, 3.960]. Moreover, the bootstrapped EGA showed that the probability of a three dimension/factor solution was = 0.682 (68.2%) and the probability of a four-dimension/factor solution was = 0.309 (30.9%).

#### Item Statistics

**FIGURE 2:**
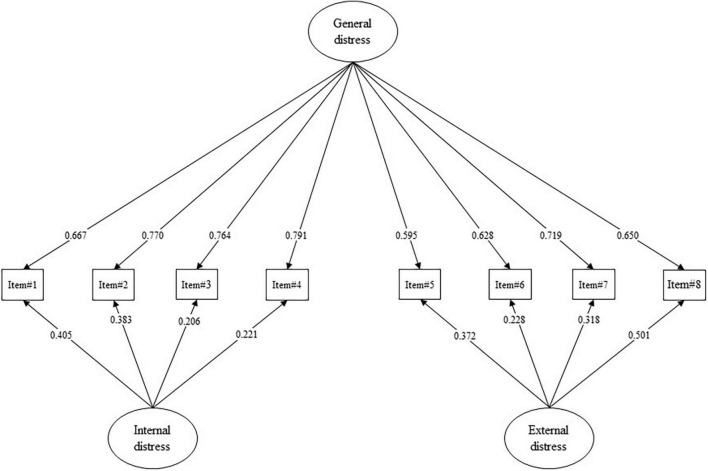
Study 2. Confirmatory factor analysis (CFA) and factor loadings. All *p*-values are less than 0.001. Absolute values of standardized factor loading (|λ|) are reported.

As reported in Table 1 and Figure 2, IS revealed that the PDI items were—on average—stable within and between their designated dimension/factor: Dim#1_IS_replication_mean_ = 0.936; Dim#2_IS_replication_mean_ = 0.708; Dim#3_IS_replication_mean_ = 0.813. More in detail, the IS analysis (Table 1) showed that most items had a good replication index. Indeed, the items in the first dimension (item#3, item#7, item#9, and item#10) displayed a replication index higher than 0.88; also the items in the second dimension (item#1, item#5, item#11, and item#13) displayed a replication index higher than 0.88. Lastly, the items in the third dimension (item#2, item#6) displayed a replication index higher than 0.99. It should be noted that item#4 (feel tired), item#8 (body image), and item#12 (sexual difficulties) did not achieve the recommended threshold of 0.80 in none of the three aforementioned dimensions.

Then, EGA-based network loadings (λ_EGA_) were computed to assess the contribution of each node to the coherence of the dimensions. As reported in Table 1, each item showed a high association with its more stable dimension/factor. Considering the first dimension/factor, λ_EGA_ ranged from 0.257 (large) to 0.426 (large). For the second dimension/factor, λ_EGA_ ranged from 0.231 (large) to 0.380 (large). For the third dimension/factor, λ_EGA_ ranged from 0.302 (large) to 0.334 (large).

Then, an in-depth examination of the semantic content of the items—grouped according to EGA—led to the labeling of the three dimensions. The first dimension measures internal states of psychological distress: anxiety (item#3), depression (item#7), lack of self-esteem (item#9), and lack of motivation (item#10). The second dimension assesses distress related to relationships and the external world: lack of desire to talk to others (item#1), loneliness (item#5), lack of external interests (item#11), worsening of relationships (item#13). Lastly, the third dimension relates exclusively to reverse items and thus a situation of wellbeing/tranquility (item#2 and Item#6).

## Study 2—Validation and an In-Depth Analysis of the Revised Version of the PDI (PDI-R)

### Materials and Methods

#### Procedure

According to the HuCARE project protocol (Passalacqua et al., 2016; Marconi et al., 2020; Caminiti et al., 2021; Rossi et al., 2021), the same procedure and inclusion/exclusion criteria of Study 1 were applied. This study was approved by the Ethics Committee of the Ospedale di Saronno (protocol n° 23247). All procedures were in accordance with the ethical standards of the institutional and/or national research committee and with the 1964 Helsinki Declaration and its later amendments or comparable ethical standards.

Also in this study, oncological outpatients of the study were still consecutively recruited at the Department of Medical Oncology, Presidio Ospedaliero di Saronno, ASST Valle Olona, in Saronno (Italy).

#### Participants

An initial sample of 936 oncological outpatients was assessed. However, 34 subjects were excluded from the sample due to missing data/answers. Therefore, the final sample comprised 902 oncological outpatients: 487 males (54%) and 415 females (46%), aged from 31 to 89 years (*mean* = 68.39, *SD* = 9.073). Considering the type of cancer, 34.8% patients had breast cancer, 24.9% patients had lung cancer, 18.8% patients had gastrointestinal cancer, 7.9% patients had urogenital cancer, 3.5% patients had pancreatic cancer, 2.7% patients had oncohematological cancer, and 7.4% patients had other type of cancer (e.g., head-and-neck cancer or skin cancer). Considering education level, 26.9% patients had an elementary school diploma, 32.8% patients had a middle school diploma, 30.8% patients had a high school diploma, and 9.5% patients had a bachelor/master’s degree. Considering civil status, 73% patients were either in a relationship or married, 6.8% patients were either separated or divorced, 10.3% patients were widowed, and 9.9% patients were single. Considering working status, 58.8% patients were retired, 20.7% patients were dependent workers, 9.6% patients were entrepreneurs, 5.6% patients were housewifes, 3.8 patients were unemployed, and 1.5% patients declared “other.”

#### Sample Size Calculation

Since the present study aimed to assess psychometric properties of a (“new”) questionnaire, the “*n:q* criterion” was used to determine the minimum sample size. In this formula, *n* is the number of subjects and *q* is the number of (free) model parameters to be estimated (Hu and Bentler, 1999; Muthén and Asparouhov, 2002; Yu, 2002). A ratio of five subjects per parameter (10:1; *n*_minimum_ = 480) was guaranteed (Bentler and Chou, 1987; Marsh et al., 1988; Hu and Bentler, 1999; Boomsma and Hoogland, 2001; Muthén and Asparouhov, 2002; Yu, 2002; Flora and Curran, 2004; Tomarken and Waller, 2005).

#### Measures

##### Psychological Distress Inventory—Revised

On the basis of the results of Study 1 and following consolidated methodological procedures (Pietrabissa et al., 2020a,b; Rossi et al., 2021), an in-depth analysis of items was performed. This procedure led to the removal of both the two reversed scored items of the original PDI (item#2 and item#6), and the three items that did not achieve IS threshold of 0.80 (item#4, item#8, and item#12). Consequently, on the basis of the results of Study 1, only two dimensions were retained: (A) *“internal”* and (B) *“external”* distress. The first dimension assessed internal states of psychological distress such as anxiety, depression, and lack of self-esteem. The second dimension assesses distress related to relationships and the external world: lack of desire to talk to others, loneliness, and worsening of relationships. Furthermore, considering the semantic content of the items, a hierarchical *“general distress”* dimension was also hypothesized. It is important to underline that the semantic content of the items has not been changed compared to the original version of the PDI provided by Morasso and colleagues (Morasso et al., 1996).

As for the original version of the PDI, the PDI-R relies on a 5-point Likert-type response scale (from 1 = “*not at all*” to 5 = “*very much*”) see Supplementary Appendix A (English version) and Supplementary Appendix B (Italian version). High scores correspond to a high degree of distress perceived by the subject.

##### The Hospitalized Anxiety and Depression Scale

The Hospitalized Anxiety and Depression Scale (HADS) was used to measure the degree of psychological suffering in oncological patients (Zigmond and Snaith, 1983; Annunziata et al., 2011; Iani et al., 2014). It is a self-report questionnaire—with a bi-factorial structure—aimed at detecting anxiety and depression in hospitalized individuals (as well as outpatients) affected by organic pathology, as well as their perceived distress as the sum of the scores obtained on the anxiety and depression dimensions (Iani et al., 2014). The HADS comprises a total of 14 items—with good reliability properties—scored on a 4-point Likert-type response scale. High scores correspond to a high degree of anxiety, depression, and distress perceived by the subject. Two cut-offs threshold for the HADS total score (HADS-T; ≥ 16 = moderate distress; ≥ 22 = severe distress) were deemed appropriate in general clinical health settings to distinguish between individuals with no-psychological suffering and those with psychological suffering (Olssøn et al., 2005; Singer et al., 2009; Vodermaier et al., 2009; Stern, 2014). In this study, Cronbach’s alpha was equal to: 0.836 for the anxiety subscale (HADS-A); 0.806 for the depression subscale (HADS-D); and 0.887 for the scale’s total score (HADS-T).

##### The Distress Thermometer

The Distress Thermometer (DT) (Bulli et al., 2008; O’Donnell, 2013; Cutillo et al., 2017) is one of the most worldwide used instruments for the measurement of emotional distress in different contexts including oncological settings. The DT is a self-report single-item measure scored on a 10-point Likert scale (from 0 = “*no distress*” to 10 = “*extreme distress*”) aimed at detecting psychological and emotive suffering. High scores correspond to a high degree of emotional distress perceived by the subject. Moreover, a cut-off threshold of ≥ 5 (Gil et al., 2005; Grassi et al., 2009; Vodermaier et al., 2009; Donovan et al., 2014) for the DT was deemed appropriate in general clinical health settings to distinguish between individuals with no-psychological distress and those who suffering from psychological distress (Grassi et al., 2009).

#### Statistical Analysis

R software (R Core Team, 2014, 2017) was used to perform statistical analyses with the following packages: cNORM (Lenhard W. et al., 2018); corrplot (Wei and Simko, 2017); irr (Gamer et al., 2019); lavaan (Rosseel, 2012; Rosseel et al., 2015), plotROC (Sachs, 2017); pROC (Robin et al., 2011), psych (Revelle, 2018), psychTools (Revelle, 2020), and semTools Contributors (2016).

A bi-factor model was specified: 4 items loaded onto the “*internal distress*” latent factor, while 4 items loaded onto the “*external distress*” latent factor, and each item also loaded onto a hierarchical “*general distress*” dimension.

The diagonal weighted least square (DWLS) estimator was used to assess the factorial structure of the PDI-R (Brown, 2015; Kline, 2016; Lionetti et al., 2016). Model fit was assessed by means of the Satorra-Bentler Chi-square statistics (S-Bχ^2^), the Root-Mean Square Error of Approximation (RMSEA), the Comparative Fit Index (CFI), and the Standardized Root Mean Residual (SRMR) (Muthén and Muthén, 1998–2017; Hoyle, 2012; van de Schoot et al., 2012; Brown, 2015; Kline, 2016). Moreover, the following cut-off criteria were chosen to evaluate the goodness of fit: (A) statistically non-significance of the χ^2^, (B) an RMSEA lower than 0.08, (C) a CFI higher than 0.95, and (D) an SRMR lower than 0.08 (Muthén and Muthén, 1998–2017; Hoyle, 2012; van de Schoot et al., 2012; Brown, 2015; Kline, 2016).

In addition, for a comprehensive evaluation of the factorial structure of the PDI-R, two alternative models were further specified and compared. First, a single factor model was specified: all of the 8 items loaded onto a single distress latent factor. Second, a first-order two-factor model was specified: 4 items loaded onto the “*internal distress*” latent factor, while 4 items loaded onto the “*external distress*” latent factor; thus, the general distress dimension was not specified.

Model evaluations were performed by using the test differences in three fit indices, with the following criteria as cutoffs for model equality: DIFFTEST (equal to Δχ^2^; *p*-value > 0.050) and ΔCFI (<0.010) (Cheung and Rensvold, 2002; Millsap and Yun-Tein, 2004; Millsap, 2012). The crossing of the cutoff of two out of three of these indices is evidence of model inadequateness.

Moreover, since the PDI-R is a new questionnaire, items’ ability to discriminate subjects with low or high *internal, external*, and *general distress* was tested (Ebel, 1965; Chiorri, 2011). Item discriminant power (IDP) was computed. More in detail, the maximum total score and quartile rank for each subject were calculated. Subsequently, a series of independent sample *t*-tests—and their effect size (Cohen’s *d*) (Cohen, 1988)—were calculated to assess item discriminating power by using the total score of the scale as a dependent variable and its lowest and highest quartile as grouping variable (Ebel, 1965; Chiorri, 2011). Moreover, item-total correlation (adjusted; i.e., *r*_it–tot_) was also computed (Howell, 2013; Pallant, 2013; Tabachnick and Fidell, 2014).

The internal consistency of each factor was evaluated with Cronbach’s alpha and McDonald’s omega (McDonald, 1999; Howell, 2013). Convergent validity was assessed with the Pearson correlation coefficient (Tabachnick and Fidell, 2014) and interpreted using the aforementioned Cohen’s benchmarks (Cohen, 1988).

Measurement invariance (MI) analyses were also performed to evaluate whether the factorial structure of the PDI-R was invariant between gender (male vs. female) and age (≤ 64 vs. ≥ 65) (Vandenberg and Lance, 2000). According to Meredith (1993) and Millsap (2012), the model structure was tested on each sample independently (Meredith, 1993). If the model fit was adequate in each sample, four nested models were sequentially specified and constrained to equality: the factorial structure (Model 1: Configural Invariance); the factorial structure and item factor loadings (Model 2: Metric Invariance); the factorial structure, item factor loadings, and item thresholds (Model 3: Scalar Invariance); the factorial structure, item factor loadings, item thresholds, and latent means (Model 4: Scalar Invariance); (Meredith, 1993; Vandenberg and Lance, 2000; Millsap, 2012). MI was assessed by using the above-mentioned test differences for model comparisons (Cheung and Rensvold, 2002; Millsap, 2012; van de Schoot et al., 2012).

Test-retest reliability of each scale was estimated on a subsample of 40 oncological patients by using the two-way mixed intraclass correlation coefficient (ICC_consistency_) (de Vet et al., 2006; Berchtold, 2016; Koo and Li, 2016).

Considering that the PDI-R (as well as the PDI) was conceptualized as a screening tool, a Receiver Operating Characteristics (ROC) curve methodology was used to assess the PDI-R accuracy to discriminate between *non*-distressed and distressed patients (Zhou et al., 2002; Pepe, 2003; Ising et al., 2012; Savill et al., 2018). More in detail, the HADS-T score cut-offs (HADS-T ≤ 15 vs. HADS-T ≥ 16 and HADS-T ≤ 21 vs. HADS-T ≥ 22) and the DT cut-off (DT ≤ 4 vs. DT ≥ 5) were used as external criterion variable and the PDI-R total score was used as the dependent variable. The global accuracy-validity of the PDI-R was estimated with the area under the ROC curve (AUC; 5000 stratified bootstrap resamples)—interpreted using the Swets’ benchmarks: AUC = 0.50, null; AUC from 0.51 to 0.70, small; AUC from 0.71 to 0.90, moderate; AUC from 0.91 to 0.99, high; and AUC = 1.00, perfect accuracy (Zweig and Campbell, 1993; Swets, 1998). Moreover, sensibility (Se) and specificity (Sp) were computed (Zhou et al., 2002; Pepe, 2003).

Lastly, as a supplemental analysis, according to previous studies procedures (Dakanalis et al., 2013), a “general sample” of patients with cancer was created by merging the sample of Study 1 and the sample of Study 2 (*N*_total_ = 1153). Thus, according to the procedure described by Gary et al. (2021), normative scores (T-scores) of the PDI-R were computed as well as the distribution percentiles of its total score (Lenhard A. et al., 2018; Gary et al., 2021).

### Results

#### Structural Validity

The PDI-R showed an excellent fit to the data. The Chi-square statistic resulted to be not statistically significant: S-Bχ^2^ (12) = 17.913; *p* = 0.118. Moreover, all the other fit indices revealed a good fit to the data: the RMSEA = 0.023; 90%CI [0.000, 0.044]; *p*(RMSEA < 0.05) = 0.984, the CFI = 0.999, the SRMR = 0.022. As reported in Table 2 and Figure 2, all the items’ loadings were statistically significant and ranged from | 0.206| (item#3, internal distress) to | 0.791| (item#4, general distress).

**TABLE 2:** Study 2. Item descriptive statistics, item psychometric properties, and factor loadings (λ) of the confirmatory factor analysis (CFA).

	Descriptive statistics				IDP		*Adj r*_(it–tot)_			CFA		
	Mean	*SD*	SK	K	*t*	*d*	Internal	External	General	Internal	External	General
Item#1	2.53	1.099	0.420	–0.441	–32.29	3.24	0.558		0.544	0.405		0.667
Item#2	2.29	1.079	0.565	–0.343	–42.33	4.22	0.638		0.560	0.383		0.770
Item#3	1.74	1.065	1.321	0.825	–23.69	2.34	0.547		0.530	0.206		0.764
Item#4	1.77	0.969	1.146	0.674	–25.93	2.57	0.575		0.651	0.221		0.791
Item#5	1.72	0.992	1.280	0.766	–24.78	2.31		0.532	0.589		0.372	0.595
Item#6	1.66	1.011	1.500	1.460	–20.34	1.89		0.475	0.618		0.228	0.628
Item#7	1.73	1.067	1.385	0.944	–26.37	2.45		0.591	0.649		0.318	0.719
Item#8	1.76	1.051	1.250	0.628	–30.97	2.88		0.609	0.608		0.501	0.650

*All test are statistically significant with p < 0.001. Skwn, Skewness; K, kurtosis; IDP, item discriminant power; t, t-test; d, Cohen’s d; Adj r_(it–tot)_, item-total correlation (adjusted).*

Moreover, this factor solution was compared with different competing models (Muthén and Muthén, 1998–2017; Brown, 2015). As reported in Table 3, model comparisons revealed the superiority of the proposed solution. Consequently, the bi-factor model solution was maintained to perform the following analysis.

**TABLE 3:** Study 2. Model comparison.

	S-Bχ^2^ (*df*)	RMSEA	CFI	Comparison	DIFF-TEST	|ΔCFI|
Model 1: bi-factor model	17.913 (12)	0.023	0.999			
Model 2: single factor model	175.593 (20)	0.082	0.987	2 vs. 1	157.68***	0.013
Model 3*:* two factors model	112.556 (19)	0.074	0.990	3 vs. 1	94.64***	0.010

***p < 0.001. S-Bχ^2^, Satorra-Bentler scaled chi-square test; df, degrees of freedoms; |Δ(…)|, absolute value of the differences between indices; RMSEA, root mean square error of approximation; CFI, comparative fit index.

#### Psychometrics Properties

The IDP analysis showed that 8 items of the PDI-R discriminated well between subjects with low or high internal and external distress (Table 2). The discrimination parameter *t*_i_ ranged from |20.34| (item#6—*external distress*) to |42.33| (item#2—*internal distress*), with an associated effect size (Cohen’s *d*) ranging from 1.89 to 4.22, respectively. Also, the item-total correlation (adjusted) revealed a moderate-to-strong association between each item and the PDI-R scores.

Reliability analysis revealed satisfying results. Indeed, for the *internal distress* subscale, the Cronbach’s alpha was equal to 0.776 and the McDonald’s omega was equal to 0.842. The *external distress* subscale showed a Cronbach’s alpha equal to 0.754 and a McDonald’s omega was equal to 0.800. The *general distress* scale showed a Cronbach’s alpha was equal to 0.853 and a McDonald’s omega was equal to 0882.

Large correlations were found between the *internal distress* scale and the HADS-A scale (*r* = 0.724, *p* < 0.001); the HADS-D scale (*r* = 0.630; *p* < 0.001), the HADS-T (*r* = 0.741; *p* < 0.001), and the DT (*r* = 0.595, *p* < 0.001). Also, moderate-to-large correlations were found between the *external distress* scale and the HADS-A scale (*r* = 0.583, *p* < 0.001); the HADS-D scale (*r* = 0.665; *p* < 0.001), the HADS-T (*r* = 0.681; *p* < 0.001), and the DT (*r* = 0.490, *p* < 0.001). Lastly, large correlations were found between the *general distress* scale and the HADS-A scale (*r* = 0.713, *p* < 0.001); the HADS-D scale (*r* = 0.705; *p* < 0.001), the HADS-T (*r* = 0.775; *p* < 0.001), and the DT (*r* = 0.576, *p* < 0.001). In addition, the internal and external distress subscales revealed a large correlation: *r* = 0.685, *p* < 0.001.

Test-retest reliability showed satisfying results: the two-way mixed ICC was equal to 0.647, 95%CI [0.385, 0.797], for the internal distress scale, to 0.699, 95%CI [0.476, 0.827], for the external distress scale, and 0.685, 95%CI [0.452, 0.819], for the general distress scale.

#### Measurement Invariance

##### Gender (Male vs. Female)

###### Model Male

The Chi-square statistic resulted to be not statistically significant: S-Bχ^2^ (12) = 13.490; *p* = 0.334. Moreover, all the other fit indices revealed a good fit to the data: the RMSEA = 0.016; 90%CI [0.000, 0.050]; *p*(RMSEA < 0.05) = 0.948, the CFI = 1.000, the SRMR = 0.025.

###### Model Female

The Chi-square statistic resulted to be not statistically significant: S-Bχ^2^ (12) = 10.860; *p* = 0.541. Moreover, all the other fit indices revealed a good fit to the data: the RMSEA = 0.000; 90%CI [0.000, 0.046]; *p*(RMSEA < 0.05) = 0.968, the CFI = 1.000, the SRMR = 0.026.

###### Configural Invariance

The configural invariance model showed good model fit indices: S-Bχ^2^ (24) = 24.350, *p* = 0.442; the RMSEA = 0.006; the CFI = 1.000; and the SRMR = 0.025; suggesting that the factor structure was similar between males and females.

###### Metric Invariance

The metric invariance model well-fitted the data: S-Bχ^2^ (40) = 77.762, *p* < 0.001; the RMSEA = 0.046, the CFI = 0.996, and the SRMR = 0.043. A statistically significant decrease in chi-square was found: DIFTEST (16) = 53.412; *p* < 0.001. However, a non-statistically significant decreases in CFI was found: |ΔCFI| = 0.004, indicating that items were equivalently related to the latent factor independently from gender.

###### Scalar Invariance

The scalar invariance model showed good model fit indices: S-Bχ^2^ (61) = 78.150, *p* < 0.001; the RMSEA = 0.025, the CFI = 0.998; and the SRMR = 0.030. A non-statistically significant decrease in chi-square was found: DIFTEST (21) = 0.387; *p* = 1. Moreover, a non-statistically significant decreases in CFI was found: |ΔCFI| = 0.002, suggesting that males and females had the same expected item response at the same absolute level of the trait.

###### Latent Means Invariance

The latent mean invariance model well-fitted the data: S-Bχ^2^ (64) = 103.994; the RMSEA = 0.037, the CFI = 0.996; and the SRMR = 0.033. A statistically significant decrease in chi-square was found: DIFTEST (3) = 25.844; *p* < 0.001. Moreover, a non-statistically significant decreases in CFI was found: |ΔCFI| = 0.002, suggesting that males and females had the same expected latent mean of the traits.

##### Age (≤ 64 y.o. vs. ≥ 65 y.o.)

###### Model ≤ 64 y.o

The Chi-square statistic resulted to be not statistically significant: S-Bχ^2^ (12) = 10.496; *p* = 0.573. Moreover, all the other fit indices revealed a good fit to the data: the RMSEA = 0.000; 90%CI [0.000, 0.051]; *p*(RMSEA < 0.05) = 0.947, the CFI = 1.000, the SRMR = 0.029.

###### Model ≥ 65 y.o

The Chi-square statistic resulted to be not statistically significant: S-Bχ^2^ (12) = 11.080; *p* = 0.552. Moreover, all the other fit indices revealed a good fit to the data: the RMSEA = 0.000; 90%CI [0.000, 0.040]; *p*(RMSEA < 0.05) = 0.990, the CFI = 1.000, the SRMR = 0.021.

###### Configural Invariance

The configural invariance model showed good model fit indices: S-Bχ^2^(24) = 21.577; the RMSEA = 0.000; the CFI = 1; and the SRMR = 0.023; suggesting that the factor structure was similar across age.

###### Metric Invariance

The metric invariance model well-fitted the data: S-Bχ^2^ (40) = 67.042; the RMSEA = 0.039, the CFI = 0.997, and the SRMR = 0.040. A statistically significant decrease in chi-square was found: DIFTEST (16) = 45.465; *p* < 0.001. However, a non-statistically significant decreases in CFI was found: |ΔCFI| = 0.003, indicating that items were equivalently related to the latent factor independently from age.

###### Scalar Invariance

The scalar invariance model showed good model fit indices: S-Bχ^2^ (61) = 68.991; the RMSEA = 0.017, the CFI = 0.999; and the SRMR = 0.032. A non-statistically significant decrease in chi-square was found: DIFTEST (21) = 1.949; *p* = 1. Moreover, a non-statistically significant decreases in CFI was found: |ΔCFI| = 0.002, suggesting that younger (≤ 64 y.o.) and older (≥ 65 y.o.) patients had the same expected item response at the same absolute level of the trait.

###### Latent Means Invariance

The latent mean invariance model well-fitted the data: S-Bχ^2^ (64) = 73.815; the RMSEA = 0.018, the CFI = 0.999; and the SRMR = 0.033. A non-statistically significant decrease in chi-square was found: DIFTEST (3) = 4.825; *p* = 0.185. Moreover, a non-statistically significant decreases in CFI was found: |ΔCFI| = 0.000, suggesting younger (≤64 y.o.) and older (≥65 y.o.) patients had the same expected latent mean of the traits.

#### Accuracy of the Psychological Distress Inventory—Revised as a Screening/Diagnostic Tool

**FIGURE 3:**
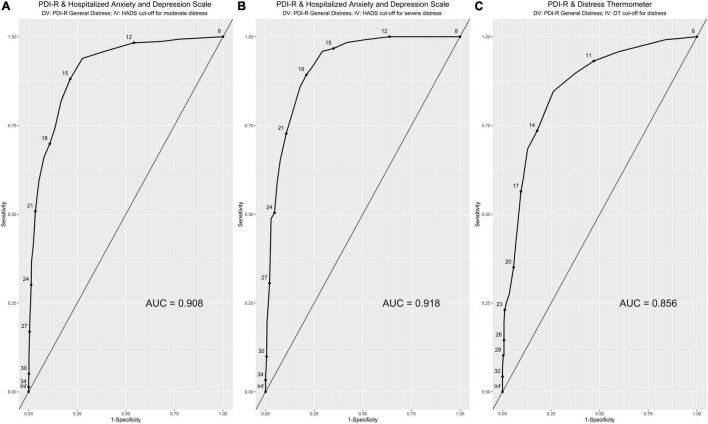
Study 2. ROC curves analysis. HADS cut-offs (**A:** moderate distress; **B:** severe distress) and the DT cut-off **(C)**. x-axis: 1-specificity; y-axis: sensitivity; AUC = area under the (ROC) curve.

Based on the HADS-T scale cut-off for moderate distress (HADS-T ≤ 15 vs. HADS-T ≥ 16), the *“general distress”* scale of the PDI-R obtained excellent accuracy in discriminating between patients without distress and patients with distress: AUC = 0.908, 95%CI [0.889, 0.928] (Table 4 and Figure 3A). Considering a cut-off point of 15 (i.e., PDI-R ≥ 15: risk of moderate distress), ROC curve revealed a SE equal to 0.881, 95%CI [0.884, 0.918], a SP equal to 0.786, 95%CI [0.753–0.818], and an ACC equal to 0.817, 95%CI [0.817, 0.817]. On the basis of the gold standard test (HADS-T; moderate distress), 67.3% of patients were classified as non-distressed and 32.4% of patients were classified as distressed. Thus, using the reported cut-off for the PDI-R, the ROC curve showed that 52.88% of individuals were correctly classified as “true negative” and 28.82% as “true positive” with a percentage of correct classification equal to 81.7%. On the contrary, 3.88% resulted to be “false negative” and 14.41% resulted to be “false positive” (18.9% of misclassification).

**TABLE 4:** Study 2. Results of the ROC analysis.

	HADS—moderate distress				HADS—severe distress				DT—general distress			
	Thr.	Sens.	Spec.	Acc	Thr.	Sens.	Spec.	Acc	Thr.	Sens.	Spec.	Acc
2	9	0.997	0.107	0.398	9	1.000	0.084	0.207	9	0.991	0.163	0.419
3	10	0.993	0.222	0.474	10	1.000	0.175	0.286	10	0.957	0.405	0.576
4	11	0.986	0.320	0.538	11	1.000	0.253	0.354	11	0.932	0.530	0.654
5	12	0.983	0.458	0.630	12	1.000	0.362	0.448	12	0.898	0.625	0.709
6	13	0.959	0.606	0.722	13	0.992	0.485	0.553	13*	**0.847**	**0.739**	**0.772**
7	14	0.939	0.723	0.794	14	0.983	0.583	0.636	14	0.737	0.822	0.796
8	15*	**0.881**	**0.786**	**0.817**	15	0.967	0.650	0.693	15	0.686	0.871	0.814
9	16	0.820	0.832	0.828	16	0.959	0.708	0.742	16	0.602	0.894	0.804
10	17	0.742	0.865	0.825	17	0.917	0.757	0.778	17	0.568	0.905	0.801
11	18	0.698	0.891	0.828	18*	**0.893**	**0.790**	**0.804**	18	0.483	0.920	0.785
12	19	0.661	0.921	0.836	19	0.859	0.822	0.827	19	0.424	0.931	0.775
13	20	0.593	0.947	0.831	20	0.794	0.858	0.849	20	0.356	0.943	0.762
14	21	0.508	0.965	0.816	21	0.727	0.894	0.871	21	0.280	0.966	0.754
15	22	0.417	0.975	0.793	22	0.653	0.924	0.888	22	0.263	0.977	0.756
16	23	0.366	0.985	0.783	23	0.587	0.941	0.894	23	0.237	0.989	0.756
17	24	0.302	0.987	0.763	24	0.504	0.954	0.893	24	0.203	0.992	0.749
18	25	0.254	0.990	0.749	25	0.488	0.972	0.907	25	0.186	0.992	0.743
19	26	0.210	0.993	0.737	26	0.388	0.976	0.897	26	0.152	0.992	0.733
20	27	0.169	0.995	0.725	27	0.306	0.979	0.889	27	0.136	0.992	0.728
21	28	0.132	0.997	0.714	28	0.248	0.986	0.887	28	0.110	0.992	0.720
22	29	0.098	0.998	0.704	29	0.198	0.992	0.886	29	0.110	0.996	0.722
23	30	0.051	0.998	0.688	30	0.099	0.995	0.875	30	0.076	1.000	0.715
24	31	0.024	1.000	0.681	31	0.050	0.999	0.871	31	0.059	1.000	0.709
25	33	0.020	1.000	0.680	33	0.050	1.000	0.872	33	0.051	1.000	0.707
26	35	0.014	1.000	0.677	35	0.033	1.000	0.870	35	0.034	1.000	0.701
27	36	0.010	1.000	0.676	36	0.025	1.000	0.869	36	0.025	1.000	0.699
28	39	0.007	1.000	0.675	39	0.016	1.000	0.868	39	0.017	1.000	0.696

*Highest average of sensitivity and specificity. Thr, Threshold; Sesn, Sensitivity; Spec, Specificity; Acc, Accuracy. HADS cut-offs (moderate distress and severe distress) and the DT cut-off. In bold are reported the highest average of sensitivity and specificity.

Based on the HADS-T scale cut-off for severe distress (HADS-T ≤ 21 vs. HADS-T ≥ 22), the *“general distress”* scale of the PDI-R obtained excellent accuracy in discriminating between patients without distress and patients with distress: AUC = 0.918, 95%CI [0.896, 0.939] (Table 4 and Figure 3B). Considering a cut-off point of 18 (i.e., PDI-R ≥ 18: risk of severe distress), ROC curve revealed a SE equal to 0.893, 95%CI [0.837, 0.948], a SP equal to 0.790, 95%CI [0.761, 0.819], and an ACC equal to 0.804, 95%CI [0.803, 0.804]. On the basis of the gold standard test (HADS-T; severe distress), 86.6% of patients were classified as non-distressed and 13.4% of patients were classified as distressed. Thus, using the reported cut-off for the PDI-R, the ROC curve showed that 68.4% of individuals were correctly classified as “true negative” and 11.97% as “true positive” with a percentage of correct classification equal to 80.37%. On the contrary, 1.44% resulted to be “false negative” and 18.18% resulted to be “false positive” (19.2% of misclassification).

Based on the DT scale cut-off for distress (DT ≤ 4 vs. DT ≥ 5), the *“general distress”* scale of the PDI-R obtained good accuracy in discriminating between patients without distress and patients with distress: AUC = 0.857, 95%CI [0.817, 0.897] (Table 4 and Figure 3C). Considering a cut-off point of 13 (i.e., PDI-R *geq* 13: risk of distress), ROC curve revealed a SE equal to 0.847, 95%CI [0.783, 0.912], a SP equal to 0.739, 95%CI [0.686, 0.792], and an ACC equal to 0.772, 95%CI [0.771, 0.773]. On the basis of the gold standard test (DT), 69.3% of patients were classified as non-distressed and 30.7% of patients were classified as distressed. Thus, using the reported cut-off for the PDI-R, the ROC curve showed that 51.05% of individuals were correctly classified as “true negative” and 26.18% as “true positive” with a percentage of correct classification equal to 77.23%. On the contrary, 4.71% resulted to be “false negative” and 18.06% resulted to be “false positive” (22.77% of misclassification).

#### Normative Scores

On the total sample of 1,153 patients with cancer (sample of Study 1 *plus* sample of Study 2), both the normative scores (T-score: *mean* = 50, *SD* = 10) and the percentile distribution were calculated. Results are reported in Tables 5, 6.

**TABLE 5:** Study 1 *plus* Study 2. Normative scores (T-scores) and percentile distribution.

Overall			Gender				Age			
*N* = 1153			Male		Female		≤64 y.o.		≥ 65 y.o.	
Raw	T	P._le_	T	P._le_	T	P._le_	T	P._le_	T	P._le_
8	31	3	32	3	32	4	32	3	33	4
9	39	14	40	15	38	11	36	8	39	14
10	43	24	43	25	42	22	40	17	42	22
11	45	33	46	33	45	32	44	27	45	30
12	48	41	48	41	48	41	46	36	47	37
13	50	48	49	48	50	49	48	44	48	44
14	51	55	51	54	52	56	50	51	50	51
15	53	61	53	60	53	63	52	58	52	57
16	54	67	54	66	55	68	54	64	53	62
17	56	71	55	70	56	73	55	69	54	67
18	57	76	57	75	57	77	56	73	56	72
19	58	80	58	78	59	81	58	77	57	76
20	59	83	59	82	60	84	59	81	58	80
21	61	86	60	85	61	87	60	84	60	83
22	62	88	61	87	62	89	61	86	61	86
23	63	90	62	89	63	91	62	89	62	88
24	64	92	63	91	65	93	63	91	63	91
25	65	94	65	93	66	95	64	92	64	92
26	66	95	66	94	67	96	65	94	65	94
27	67	96	67	95	68	97	66	95	67	95
28	69	97	68	96	70	98	69	96	68	96
29	70	98	69	97	71	98	69	97	69	97
30	71	98	70	98	73	99	70	98	70	98
31	72	99	71	98	76	99	72	98	71	98
32	73	99	72	99	78	99	73	99	72	99
33	75	99	73	99			75	99	74	99
34	76	99	74	99			79	99	75	99
35	78	99	76	99			79	99	76	99
36	80	99	77	99			79	99	77	99
37	80	99	78	99			79	99	79	99
38	80	99	80	99					80	99
39	80	99	80	99					80	99
40	80	99	80	99					80	99

*Raw, General distress (PDI-R) raw score; T, T-score; P._le_, percentile. Overall sample, N = 1153; Male, n = 487; Female, n = 415; = 64 y.o.: n = 327; = 65 y.o.: n = 63.7.*

**TABLE 6:** Study 1 *plus* Study 2. Normative scores (T-scores) and percentile distribution for gender and age.

Male					Female			
≤ 64 y.o.			≥ 65 y.o.		≤ 64 y.o.		≥ 65 y.o.	
Raw	T	P._le_	T	P._le_	T	P._le_	T	P._le_
8	33	3	34	5	32	4	32	3
9	36	8	40	16	36	9	37	9
10	40	16	43	24	41	18	41	18
11	43	24	45	32	45	30	44	26
12	45	32	47	39	48	41	46	35
13	47	39	49	46	50	50	48	42
14	49	46	51	52	52	58	50	49
15	51	52	52	58	54	64	52	56
16	52	58	53	63	55	70	53	62
17	53	63	55	68	57	75	54	67
18	55	68	56	73	58	79	56	72
19	56	72	57	76	59	82	57	76
20	57	76	58	80	60	85	58	80
21	58	80	60	83	62	88	60	83
22	59	83	61	86	63	90	61	86
23	61	85	62	88	64	92	62	88
24	62	88	63	90	65	93	63	91
25	63	90	64	92	66	95	64	93
26	64	92	65	93	68	96	66	94
27	65	93	66	95	69	97	67	96
28	66	95	67	96	70	98	68	97
29	67	96	68	96	72	99	70	98
30	69	97	69	97			72	99
31	70	98	70	98			74	99
32	71	98	71	98			77	99
33	73	99	72	99				
34	76	99	73	99				
35	78	99	74	99				
36	78	99	75	99				
37	78	99	76	99				
38			77	99				
39			78	99				
40			79	99				

*Raw, General distress (PDI-R) raw score; T, T-score; P._le_, percentile. Male = 64 y.o.: n = 170; Male = 65 y.o.: n = 317; Female = 64 y.o.: n = 157; Female = 65 y.o.: n = 258.*

## General Discussion

In the last decades, psychological research in medical and oncological settings constantly increased focusing on observed outcomes of psychological care (Mannarini et al., 2013; Caminiti et al., 2017, 2021; Rossi et al., 2021). In this context, the construct of psychological distress has rapidly gained popularity (Mitchell, 2010; Wise et al., 2013; Riba et al., 2019; Marconi et al., 2020). Indeed, due to its severe negative impacts on medical outcomes (DiMatteo et al., 2000), distress represents one of the most important indexes of suffering in oncological patients (Mitchell, 2010; Cutillo et al., 2017). This interest led to the development of several questionnaires aimed at measure this construct (Carlson and Bultz, 2003; Herschbach et al., 2004; Mitchell, 2010; Wise et al., 2013; Cutillo et al., 2017; Riba et al., 2019).

In particular, the PDI (Morasso et al., 1996) is one of the most used instruments worldwide (Wise et al., 2013; Riba et al., 2019). The PDI was originally developed in the Italian context: it is a thirteen-item self-report instrument (Morasso et al., 1996) and it is among the most recommended tools to screen for distress in oncology (Vodermaier et al., 2009; Muzzatti and Annunziata, 2012)—however, the original validation study showed that it lacks of a rigorous assessment of its (basic) psychometric properties such as its factorial structure. Indeed, The only identified validation study of PDI concerned Italian cancer patients (Morasso et al., 1996). In this study, criterion, concurrent and discriminant—but not construct—validity were reported, together with good internal consistency (Muzzatti and Annunziata, 2012). Despite it provides encouraging, albeit incomplete, information on reliability and validity, data on construct validity are strongly requited for a screening tool (Muzzatti and Annunziata, 2012).

Starting from this background, in line with previous researches (Panzeri et al., 2021c; Parola et al., 2022; Rossi et al., 2022), the aim of the first study here reported was to extensively examine the factorial structure of the PDI. To achieve this goal, EGA was used: an innovative technique, particularly precise and sensitive in identifying the correct number of factors/dimensions (Golino and Epskamp, 2017). Moreover, on the basis of the results of Study 1, the second study (Study 2) aimed at developing a (shortened) revised version of the PDI (PDI-R) and to provide an in-depth analysis of its psychometric properties including its factorial structure and measurement invariance, thus filling the gap in the literature. To achieve this, gold-standard statistical techniques were used to analyze the psychometric properties of the questionnaires (e.g., CFA).

The first study revealed that the (original) supposed single-factor structure of the PDI (Morasso et al., 1996)—one latent dimension/factor accounting for all of the 13 items—was not adequate. Indeed, the EGA revealed that the original pool of items grouped onto three different dimensions/factors—instead of a single one (Figure 1). These dimensions refer to: (1) internal states of psychological distress such as anxiety and depression; (2) relationships and the external world: lack of desire to talk to others and loneliness; and (3) wellbeing/tranquility. It should be noted that this result might be attributed to the original structuring procedure of the PDI (Morasso et al., 1996) and the statistical analysis performed. Indeed, Morasso et al. (1996) did not test the factorial structure of the PDI but only evaluated its internal consistency—and, since Cronbach’s alpha resulted good, a single-factor structure was inferred. In addition, despite the original validation article contained three studies attempting to validate the PDI, each of them was conducted relatively small sample sizes and thus, results may be biased.

In addition, scientific literature showed that modern techniques of statistical analysis—such as EGA (Golino and Epskamp, 2017)—are more strongly adequate and precise to extract the right number of factors of a questionnaire than classical exploratory techniques (e.g., exploratory factor analysis, EFA) (Golino and Demetriou, 2017; Christensen and Golino, 2019, 2020; Christensen, 2020; Christensen et al., 2020a; Golino et al., 2020b).

Results from Study 1 shed new light on the dimensional/factor structure of the PDI—inspiring the development of its revised (shortened) version: the PDI-R (Study 2).

For the validation and study of the psychometric properties of the PDI-R redundant items were removed: in particular, the two reversed scored items (PDI: item#2 and item#6). Moreover, also the three items that did not achieve the Item Stability threshold (PDI: item#4, item#8, and item#12) were removed. Consequently, only two first-order latent dimensions were retained: internal and external distress; and a second-order general distress dimension was hypothesized. Thus the factorial structure of a new, brief but solid questionnaire was tested on a large sample of oncological outpatients.

A bi-factor structure was specified, and the CFA successfully confirmed that all the 8 items of the PDI-R loaded onto the supposed first-order latent factor (internal and external distress) and all the items loaded on the general dimension of distress (Figure 2). CFA also revealed that the PDI-R had good structural validity with good fit indices (Muthén and Muthén, 1998–2017; Hoyle, 2012; van de Schoot et al., 2012; Brown, 2015).

Moreover, considering that the PDI-R is a new scale, the proposed factorial structure was compared with possible competing models: a single-factor model and a two-first order model. Results of model comparison demonstrated that the proposed bi-factor structure is the best factorial solution for the PDI-R.

Item discrimination power was also tested. Results showed that each of the first four items composing the PDI-R well discriminated between subjects with low or high internal distress. At the same time, for the external subscale, the item discrimination power indicated that each of the second four items comprising the PDI-R well discriminated between subjects with low or high external distress. These results suggest the goodness of the items to discriminate between different types of distress in the individuals, and the ability of each item to represent its latent construct.

Reliability analyses were also performed, providing satisfying results for the internal, external, and general distress scale. In addition, the 1-month test-retest reliability provided good results – as shown by the two-way mixed ICCs (Pursey et al., 2016).

Convergent validity analyses were also performed. In line with the scientific literature, significant correlations were found between the PDI-R total score and other well-consolidated measures of psychological suffering such as the HADS and the DT (Wise et al., 2013; Riba et al., 2019). Strong correlations were found between the PDI-R general distress scale, the HADS total score (*r* = 0.775), its subscales (HADS anxiety, *r* = 0.713; HADS depression, *r* = 0.705), and the DT (*r* = 0.576). These correlations suggest a strong association of distress (measured by the PDI-R) with psychological and emotive difficulties in oncological patients—due to the possible presence of people with severe diagnoses and related preoccupations and fears in the sample (Rossi et al., 2021).

Moreover, MI was tested to explore at which level (structural vs. loadings vs. intercepts/thresholds, means) there were differences across gender (males vs. females) and age (≤ 64 vs. ≥ 65). MI analysis showed that latent means invariance was achieved. This suggests that the eight items were equivalently related to the latent distress factors across each sample, and indicates that samples had the same expected item response at the same absolute level of the trait. Thus, males and females as well as patients with different age interpreted the PDI-R items in the same way (the factorial structure was equal across samples),with the same strength (items were related to the latent construct equally between samples), with the same “starting point” (item thresholds were similar among samples) and had the same latent mean of the construct (latent means were similar across samples). Consequently, experienced psychological distress measured by the PDI-R can be evaluated and compared between males and females as well as between patients with different age (≤ 64 vs. ≥ 65).

In addition, results from the ROC analyses showed that the PDI-R is an excellent screening/diagnostic tool for the detection of psychological distress. Indeed, the PDI-R presented an excellent accuracy in discriminating between distressed and non-distressed oncological outpatients. More in detail, considering the principal aim of a screening tool—i.e., to capture the majority of “positives” patients (Zou et al., 2012)—the PDI-R showed excellent sensitivity properties. At the same time, it is important to underline that—in contrast to many screening tools—specificity is not compromised and it showed very good values. These results suggest that—in spite of the small number of items (*n* = 8)—the PDI-R is an excellent instrument for the screening of distress in samples of cancer patients. In this regard, it should be emphasized that the proposed cut-offs are those with the best average ratio between sensitivity and specificity on a sample of 902 cancer patients and should therefore be considered as guidelines for patient classification—which, however, cannot replace the clinical interview. However, these cut-offs should not be used in an absolutely inflexible way: they should be evaluated according to the situation and what is to be obtained from the screening assessment (Cohen, 1988).

Consequently, in order to guide the interpretation of the score obtained on the “general distress scale” of the PDI-R, normative scores (T-scores) and the percentiles distribution were calculated—on a large sample of 1,153 patients with cancer. Using the cut-offs that emerged from Study 2 (i.e., PDI-R ≥ 15: moderate distress; PDI-R ≥ 18: severe distress) it is possible to observe that the thresholds for moderate and severe distress are, respectively, located in the presence of the 60th and 75th percentiles—underlining the adequacy of the previously proposed guidelines.

Despite these interesting findings, several limitations have to be highlighted. First, despite the sample size being adequate to perform a CFA, the provided bi-factor model might be expensive in terms of sample-to-parameter ratio. Consequently, cross-cultural adaptation and validation studies aimed at computing the factorial structure of the PDI-R should consider enrolling an appropriate number of outpatients. Moreover, the number of participants (*n* = 40) to which the PDI-R was re-administered was enough for the assessment of the test-retest reliability but far from being adequate for a longitudinal MI analysis. In addition, the sample of the present research was only composed by oncological outpatients: future studies should investigate the factorial structure of this inventory in other categories of (hospitalized) patients.

Despite these limitations, it is also important to emphasize some strengths of the present research work. First of all, this work is based on a two-step methodology that has already been consolidated in previous research. Second, both questionnaires (i.e., PDI and PDI-R) were extensively analyzed using—for example—innovative statistics (e.g., EGA). Third, both studies are based on large sample sizes, which makes it possible to use robust and reliable statistics. Fourth, the study of factorial invariance showed that the PDI-R is an instrument that is widely applicable to heterogeneous contexts with patients of both sexes or different age groups.

Still, this contribution shows that the PDI-R might be a good instrument for the assessment of psychological distress in oncological settings and that it might also be used for research purposes.

Last, the PDI-R might also represent a starting point for the assessment of psychological distress and the planning of (psycho-)oncological treatments aimed to reduce of the individuals’ psychological suffering and the general health status of patients in oncological settings.

## Data Availability Statement

The datasets presented in this article are not readily available due to privacy restrictions, data were available from the corresponding author on a reasonable request. Requests to access the datasets should be directed to corresponding author.

## Ethics Statement

The studies involving human participants were reviewed and approved by the Ethics Committee of the Ospedale di Saronno. The patients/participants provided their written informed consent to participate in this study.

## Author Contributions

AAR conceived the study, wrote the first draft, performed statistical analyses, and displayed tables and figures. MM collected the data. FT wrote part of the first draft. CV and SM supervised the work and revised the final version of the manuscript. All authors read, approved the work, and contributed to the article and approved the submitted version.

## Conflict of Interest

The authors declare that the research was conducted in the absence of any commercial or financial relationships that could be construed as a potential conflict of interest.

## Publisher’s Note

All claims expressed in this article are solely those of the authors and do not necessarily represent those of their affiliated organizations, or those of the publisher, the editors and the reviewers. Any product that may be evaluated in this article, or claim that may be made by its manufacturer, is not guaranteed or endorsed by the publisher.
