# Supplementary material for: Screening for Distress in Oncological Patients: The Revised Version of the Psychological Distress Inventory (PDI-R)
Source: Front Psychol. 2022 May 6;13:859478. doi: 10.3389/fpsyg.2022.859478 (PMC9121122; doi:10.3389/fpsyg.2022.859478)
Supplement: Supplementary file 1 [file Data_Sheet_1.PDF]

Psychological Distress Inventory – Revised (PDI-R)

INSTRUCTIONS: Below are some questions. Your answers will help us to understand how you have been feeling over the past week. Please read carefully one question at a time and choose the answer that best describes your situation **over the past week**.

ATTENTION: please try to answer all questions as accurately as possible. THERE ARE NO RIGHT OR WRONG ANSWERS

1	2	3	4	5
NOT AT ALL	A LITTLE BIT	SOMEWHAT	QUITE A BIT	VERY MUCH

Over the past week:

1	Have you experienced moments of anxiety or inner tension?	1	2	3	4	5
2	Have you experienced moments of dejection or depression?	1	2	3	4	5
3	Have you felt worthless?	1	2	3	4	5
4	Have you felt a lack willpower?	1	2	3	4	5
5	Do you think that your desire to speak with others has diminished?	1	2	3	4	5
6	Have you felt more alone?	1	2	3	4	5
7	Has your interest in the world around you diminished?	1	2	3	4	5
8	Has the illness negatively influenced your relationships with others?	1	2	3	4	5

Rossi, A.A., Marconi, M., Taccini, F., Verusio, C. & Mannarini S. (2022). *Screening for Distress in Oncological Patients: the Revised Version of the Psychological Distress Inventory (PDI-R)*. Frontiers in Psychology.
