# Supplementary material for: Screening for Distress in Oncological Patients: The Revised Version of the Psychological Distress Inventory (PDI-R)
Source: Front Psychol. 2022 May 6;13:859478. doi: 10.3389/fpsyg.2022.859478 (PMC9121122; doi:10.3389/fpsyg.2022.859478)
Supplement: Supplementary file 2 [file Data_Sheet_2.PDF]

Psychological Distress Inventory – Revised (PDI-R)

ISTRUZIONI: Sono qui di seguito riportate alcune domande. Le sue risposte ci aiuteranno a capire meglio come si è sentito in questo ultimo periodo. La preghiamo di leggere attentamente una domanda alla volta e di scegliere la risposta che le sembra meglio descrivere la sua situazione dell'**ultima settimana**

ATTENZIONE: la preghiamo di provare a rispondere a tutte le domande nel modo più preciso possibile. NON ESISTONO RISPOSTE GIUSTE O SBAGLIATE

1	2	3	4	5
Per nulla	Poco	Abbastanza	Molto	Moltissimo

Nell'ultima settimana:

1	Ha avuto momenti di ansia, di tensione interna?	1	2	3	4	5
2	Ha avuto momenti di sconforto o depressione?	1	2	3	4	5
3	Si è sentito/a senza valore?	1	2	3	4	5
4	Si è sentito/a senza volontà?	1	2	3	4	5
5	Pensa che la sua voglia di parlare con gli altri sia diminuita?	1	2	3	4	5
6	Si è sentito/a più solo/a?	1	2	3	4	5
7	L'interesse per il mondo che la circonda è diminuito?	1	2	3	4	5
8	La malattia ha influenzato negativamente le sue relazioni con gli altri?	1	2	3	4	5

Rossi, A.A., Marconi, M., Taccini, F., Verusio, C. & Mannarini S. (2022). *Screening for Distress in Oncological Patients: the Revised Version of the Psychological Distress Inventory (PDI-R)*. Frontiers in Psychology.
